# Isotretinoin Use During Consolidation in Acute Promyelocytic Leukemia Following Standard All-Trans Retinoic Acid (ATRA)-Based Induction: A Case Report

**DOI:** 10.7759/cureus.106907

**Published:** 2026-04-12

**Authors:** Anushareddy Muddasani, Rachel J Hendrix, Jennifer Laudadio, Cesar Gentille

**Affiliations:** 1 Internal Medicine, University of Arkansas for Medical Sciences, Little Rock, USA; 2 Pharmacology, University of Arkansas for Medical Sciences, Little Rock, USA; 3 Pathology, University of Arkansas for Medical Sciences, Little Rock, USA; 4 Hematology and Oncology, University of Arkansas for Medical Sciences, Little Rock, USA

**Keywords:** acute promyelocytic leukemia, all-trans retinoic acid (atra) therapy, arsenic trioxide (ato), differentiation therapy, isotretinoin

## Abstract

Acute promyelocytic leukemia (APL) is a subtype of acute myeloid leukemia characterized by the presence of the promyelocytic leukemia-retinoic acid receptor alpha (PML::RARA) fusion gene. The combination of all-trans retinoic acid (ATRA) and arsenic trioxide (ATO) has significantly improved outcomes in patients with APL. Isotretinoin (13-cis retinoic acid), a stereoisomer of ATRA commonly used for acne vulgaris, shares a similar mechanism of action and has demonstrated differentiation-inducing activity in vitro and in limited clinical reports.

We present the case of a 38-year-old uninsured male with low-risk APL, defined by a presenting white blood cell count of ≤10,000/µL per the Sanz risk stratification system, who achieved hematologic complete remission following standard induction therapy with ATRA plus ATO but was transitioned to isotretinoin during consolidation because he was unable to obtain ATRA due to financial constraints. The patient achieved molecular complete remission within two months of initiating isotretinoin-based consolidation therapy and continues to have negative PML::RARA reverse transcriptase polymerase chain reaction (RT-PCR) results, with sustained remission at 18 months of follow-up with ongoing molecular monitoring every three months.

Although the concurrent use of ATO and prior ATRA-based induction are important confounders, this case highlights the potential role of isotretinoin as a cost-effective alternative retinoid for consolidation therapy in APL when ATRA is inaccessible, although ATRA remains the recommended standard treatment.

## Introduction

Acute promyelocytic leukemia (APL) is a distinct subtype of acute myeloid leukemia (AML) characterized by the t (15;17) (q24;q21) translocation resulting in the promyelocytic leukemia-retinoic acid receptor alpha (PML::RARA) fusion gene, which disrupts normal myeloid differentiation [1].

Historically associated with high early mortality due to severe coagulopathy, the prognosis of APL has dramatically improved with the introduction of differentiation therapy. The combination of all-trans retinoic acid (ATRA) and arsenic trioxide (ATO) has become the current standard of care for low- and intermediate-risk disease, achieving complete remission rates exceeding 90% and transforming APL into one of the most curable forms of leukemia [2].

Contemporary European LeukemiaNet (ELN) guidelines endorse ATRA plus ATO as the preferred regimen for both induction and consolidation in low-risk APL, with consolidation delivered over multiple cycles of ATRA in combination with ATO [3,4]. However, access to ATRA remains a significant real-world barrier. A national survey found that only 31% of U.S. hospitals had ATRA in stock, with availability as low as 14% among referring centers, and the cost of oral tretinoin can be prohibitive for uninsured patients, potentially compromising outcomes in an otherwise highly curable malignancy [5].

ATRA promotes differentiation of malignant promyelocytes by targeting the retinoic acid receptor alpha component of the PML-RARA fusion protein, thereby relieving the differentiation block that characterizes APL [6].

Isotretinoin (13-cis retinoic acid), a stereoisomer of ATRA widely used in dermatologic practice for the treatment of severe acne [7], differs pharmacologically from ATRA in that it has minimal direct binding affinity to nuclear retinoid receptors and cellular retinoic acid-binding proteins. Instead, isotretinoin functions largely as a prodrug, requiring intracellular isomerization to ATRA to exert its differentiation-inducing effects [8].

Despite this indirect mechanism, isotretinoin has demonstrated differentiation-inducing activity in both in vitro and in vivo studies, with limited case reports suggesting its potential as an alternative to ATRA in the treatment of APL [9-11].

Here, we present a case highlighting the use of isotretinoin as a pragmatic alternative during consolidation therapy in APL when ATRA is not accessible, adding to the limited but growing body of evidence supporting isotretinoin's biologic activity in this disease while emphasizing its potential role in addressing real-world treatment barriers.

## Case presentation

A 38-year-old man with no significant past medical or surgical history presented to our hospital with an initial diagnosis of APL based on a bone marrow biopsy performed at an outside hospital. He initially presented there with symptoms of shortness of breath, cough, and intermittent fevers lasting for one month, and was found to have pancytopenia. On arrival, he was hypoxic and placed on oxygen via nasal cannula. Physical examination was otherwise unremarkable, with no hepatosplenomegaly, lymphadenopathy, bleeding manifestations, or skin findings. His computed tomography angiography (CTA) of the chest revealed pulmonary emboli, and he was started on a heparin infusion. His chest radiograph showed signs of pneumonia; an infectious workup was ordered, and antibiotics were started. Initial blood cultures were negative.

Patient consent was waived by the institutional review board, as this case report contains no identifiable patient information.

Laboratory findings at presentation are summarized in Table 1, and the clinical timeline of diagnosis, treatment, and follow-up is outlined in Table 2.

**Table 1:** Laboratory findings at presentation. Notable findings include pancytopenia with 14% peripheral blasts. D-dimer was markedly elevated at 25.93 ng/mL, while PT (12.2 s), INR (1.1), and fibrinogen (440 mg/dL, elevated) remained within or above normal limits. This pattern of elevated D-dimer without prolongation of coagulation times or hypofibrinogenemia is consistent with APL-associated coagulopathy with predominant hyperfibrinolysis rather than overt consumptive DIC. LDH and uric acid were within normal limits, indicating low tumor lysis risk. Based on the presenting WBC of 1,100/µL and platelet count of 100,000/µL, the patient was classified as low-risk APL per Sanz risk stratification criteria. APL = acute promyelocytic leukemia; DIC = disseminated intravascular coagulation; INR = international normalized ratio; LDH = lactate dehydrogenase; PT = prothrombin time; WBC = white blood cell count

Laboratory Test	Result	Reference Range
White blood cell count	1,100 cells/µL	4,000-11,000 cells/µL
Hemoglobin	8 g/dL	13-17 g/dL
Platelet count	100,000 cells/µL	150,000-450,000 cells/µL
Prothrombin time	12.2 seconds	11-13.5 seconds
International normalized ratio (INR)	1.1	0.8-1.2
D-dimer	25.93 ng/mL	0.5 ng/mL
Fibrinogen	440 mg/dL	200-400 mg/dL
LDH	202 U/L	135-225 U/L
Uric acid	3.4 mg/dL	3.5-7.2 mg/dL
Peripheral blood blasts	14%	0%

**Table 2:** Clinical timeline of diagnosis, treatment, and follow-up.

Time	Clinical Event
Month 0	Presentation with dyspnea, pneumonia, pulmonary embolism, and pancytopenia
Day 0	Bone marrow biopsy confirming acute promyelocytic leukemia (APL)
Days 1-28	Induction therapy with all-trans retinoic acid (ATRA) and arsenic trioxide (ATO)
Day 28	Hematologic complete remission achieved
Month 1	Consolidation therapy initiated with isotretinoin (13-cis retinoic acid) and ATO
Month 3	PML-RARA PCR negative, consistent with molecular remission
Follow-up	PML-RARA PCR monitoring every three months
18 months	Patient remains in sustained molecular remission

**Figure 1:**
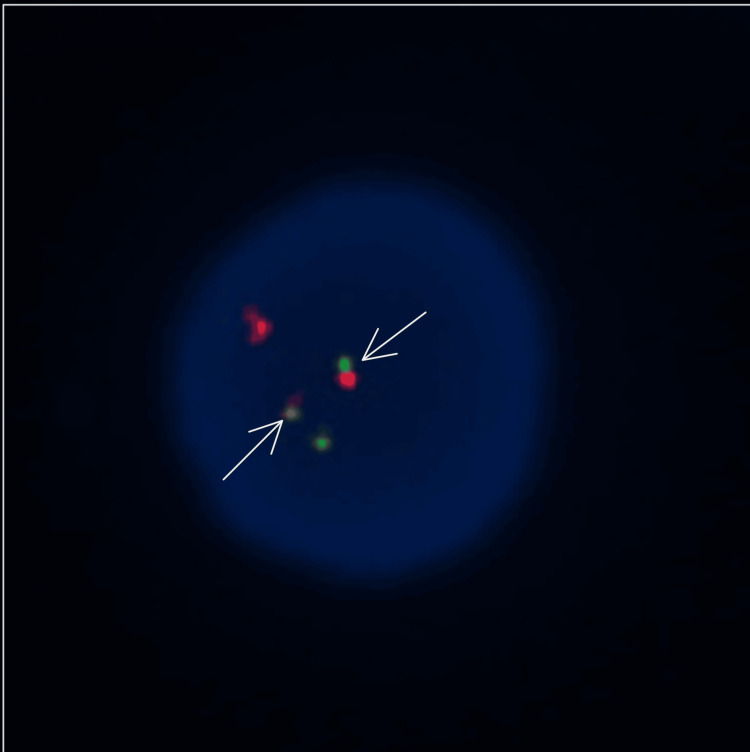
One cell showing a one red, one green, and two fusion (1R1G2F) signal pattern diagnostic of PML::RARA. Arrows point to the two fusion signals identifying the derivative chromosomes involved in the translocation. PML, spectrum red/orange probe; RARA, spectrum green probe. Fluorescence in situ hybridization (FISH) demonstrating the PML::RARA fusion signal, confirming the diagnosis of acute promyelocytic leukemia with the t (15;17) (q24;q21) translocation. The presence of this fusion is diagnostic of APL and identifies patients who benefit from targeted therapy with all-trans retinoic acid and arsenic trioxide. PML::RARA = promyelocytic leukemia-retinoic acid receptor alpha

His bone marrow biopsy showed marrow cellularity of 70% with 78% blasts/blast equivalents. Fluorescence in situ hybridization (FISH) using a dual fusion probe set confirmed the presence of the promyelocytic leukemia-retinoic acid receptor alpha (PML::RARA) t (15;17) (q24;21) translocation in 185 of 200 analyzed nuclei (92.5%). The patient was started on induction therapy with ATRA at 45 mg/m²/day for one day and was subsequently transferred to our hospital for definitive management of APL. He received 28 days of induction chemotherapy with ATRA 45 mg/m²/day plus ATO 0.15 mg/kg/day. A subsequent bone marrow biopsy showed hematologic complete response. At that time, the karyotype was normal, but FISH testing confirmed the continued presence of t (15;17) (PML::RARA) in 29 of 100 scored nuclei (29%) (Figure 1). However, persistence of cytogenetic markers after induction is expected in APL and does not carry prognostic or therapeutic implications.

**Figure 2:**
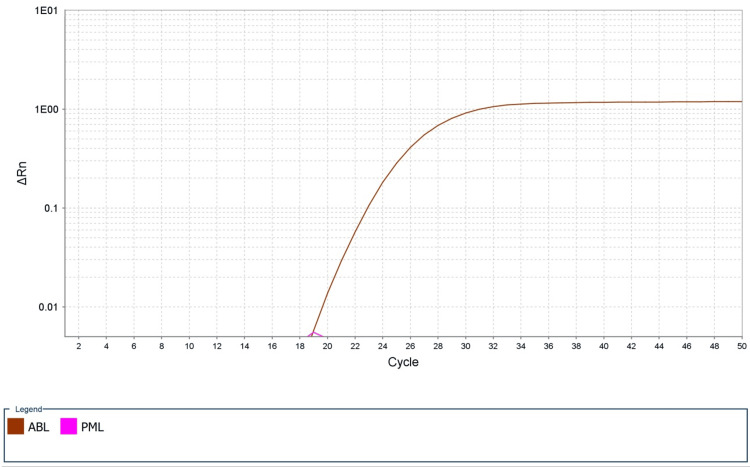
PCR cycle versus the change in fluorescence plot showing PML::RARA amplification curve. An amplification curve is detected for the internal control, ABL1 (brown), confirming sufficient RNA in the sample. No amplification curve is detected for PML::RARA (pink), indicative of no detectable fusion transcripts, confirming molecular complete remission. PML::RARA = promyelocytic leukemia-retinoic acid receptor alpha

His blood counts returned to normal levels at discharge. Due to a lack of insurance and the high cost of ATRA, he was unable to continue it at discharge. Instead, he was discharged on oral isotretinoin at 100 mg/m²/day (divided doses) for two weeks on, two weeks off, in combination with ATO for consolidation therapy. Within two months of starting this regimen, quantitative PML::RARA reverse transcriptase polymerase chain reaction (RT-PCR) was negative. The test was designed to detect long (bcr1), variable (bcr2), and short (bcr3) PML::RARA transcripts and had a 0.005% limit of detection. The internal control RNA used for normalization was detected (Figure 2).

The isotretinoin dose was then reduced to 60 mg/m²/day (divided doses) following achievement of molecular remission, as part of a step-down approach and not due to treatment-related toxicity. He completed consolidation therapy consisting of seven cycles of isotretinoin and four cycles of ATO, with ATO administered at 0.15 mg/kg/day intravenously in a cyclical schedule of four weeks on treatment followed by four weeks off for a total of four cycles. During consolidation, the patient was monitored with biweekly complete blood counts and coagulation studies. Liver function tests and lipid panels were obtained monthly, given isotretinoin's known hepatotoxic and dyslipidemic potential. No adverse effects were observed during therapy, including no mucocutaneous toxicity, hepatotoxicity, or hypertriglyceridemia, and no dose adjustments were required. At the most recent follow-up, PML::RARA RT-PCR remains negative, indicating complete molecular remission, and his blood counts are within normal range. He continues to undergo quantitative PML::RARA RT-PCR monitoring every three months and remains in remission.

## Discussion

This case highlights the use of isotretinoin as an alternative therapy for APL when ATRA is unavailable due to financial constraints. The patient initially received standard induction therapy with ATRA plus ATO for 28 days and achieved morphologic complete remission, but was subsequently transitioned to isotretinoin during consolidation due to the prohibitive cost of ATRA, as the patient was uninsured. The patient has remained in remission with persistent PML::RARA RT-PCR negativity, suggesting that isotretinoin retained sufficient retinoid activity to promote differentiation and disease control. 

Retinoid-induced differentiation is the cornerstone of APL therapy [12]. The biologic rationale for retinoid activity in leukemia was first demonstrated by Breitman et al., who showed in vitro that retinoids could induce differentiation of the HL-60 promyelocytic leukemia cell line [9]. Shortly thereafter, early clinical reports suggested that isotretinoin could induce maturation of leukemic promyelocytes in vivo. Flynn et al. reported hematologic improvement and differentiation in a chemotherapy-refractory APL patient treated with isotretinoin (100 mg/m²/day) [13], and Nilsson described probable in vivo differentiation with retinoids other than ATRA [14]. 

Importantly, these early reports occurred during a period when ATRA was not yet widely available or established as standard therapy, and conventional anthracycline-based chemotherapy remained the primary recognized treatment for APL [1]. Thus, isotretinoin use in these pioneering cases was experimental, providing early proof-of-concept that retinoid-induced differentiation was a class effect and not exclusive to ATRA. 

Only later did clinical investigators in China establish ATRA as a transformative therapy. In the landmark study by Huang et al., patients treated with ATRA achieved high complete remission rates without prolonged marrow aplasia, thereby initiating the modern era of differentiation therapy in APL [15]. These findings were subsequently confirmed in Western populations, where Warrell et al. demonstrated that ATRA induced remission through maturation of leukemic promyelocytes and linked clinical response to the aberrant retinoic acid receptor-α associated with the t (15;17) translocation [12]. Over subsequent decades, ATRA-based regimens became the global standard of care, culminating in contemporary risk-adapted approaches combining ATRA with ATO, which achieve remission rates exceeding 90% [2]. 

Modern reports of isotretinoin use occur in an era when ATRA is firmly established as the gold standard. Alkhaldy et al. observed hematologic improvement within five days of initiating isotretinoin (0.6 mg/kg/day) in a patient unable to access ATRA [10]. More recently, Goldin et al. reported molecular complete remission in a patient who inadvertently received isotretinoin (45 mg/m²/day) instead of ATRA in combination with ATO due to a prescription error [11]. These contemporary cases demonstrate the biologic activity of isotretinoin but do not redefine standard therapy; rather, they support its potential role as a temporary substitute when ATRA is inaccessible. 

The cost differential between oral tretinoin and isotretinoin is substantial. Based on current average wholesale pricing, oral tretinoin is approximately five-fold more expensive than isotretinoin on a per-capsule basis. For an uninsured patient, the monthly out-of-pocket cost of ATRA-based consolidation therapy can exceed several thousand dollars, whereas isotretinoin-based therapy costs a fraction of this amount. Moreover, ATRA availability is a recognized systemic challenge in the United States; a national survey by Geer et al. found that only 31% of surveyed hospitals had ATRA in stock, with availability as low as 14% among referring centers [5]. These findings underscore that the barrier to ATRA access is not solely financial but also logistical, and that alternative retinoid strategies may be clinically necessary in resource-constrained settings. While patient assistance programs and generic formulations exist, they may not be accessible to all patients in real-time clinical scenarios. Isotretinoin, widely available as a generic medication for dermatologic indications, represents a substantially more affordable alternative that may bridge this access gap. 

The successful outcome in this case, achieving and maintaining molecular remission, suggests that isotretinoin may provide adequate retinoid activity for consolidation therapy when combined with ATO, particularly in patients who have already achieved hematologic remission with standard ATRA-based induction. However, this observation must be interpreted cautiously, given the synergistic role of ATO in APL therapy. 

Of additional clinical interest, this patient presented with pulmonary embolism at diagnosis. Although hemorrhagic complications are the hallmark of APL-associated coagulopathy, thrombotic events are increasingly recognized as significant manifestations of APL-associated coagulopathy. The PETHEMA/PALG registry (n = 1,210) reported thrombo-ischemic events in 16% of APL patients, with PE occurring in 2.1% [16]. Mitrovic et al. found a thrombotic event rate of 20.6%, with increased detection when systematic D-dimer monitoring was implemented, suggesting historical underdiagnosis [17]. The pathophysiology underlying this prothrombotic state includes tissue factor overexpression, cancer procoagulant release, TF-bearing microparticles, and inflammatory cytokine secretion by leukemic promyeloblasts, which collectively drive excessive thrombin generation [18]. The occurrence of PE in our patient likely reflects this APL-driven coagulopathy rather than an incidental event, given the timing at diagnosis and absence of traditional venous thromboembolism (VTE) risk factors. 

An important limitation of this case is the potential confounding effect of ATO. ATO has well-established independent efficacy in APL and has been shown to induce durable remissions, including in chemotherapy-free regimens. Because isotretinoin was administered in combination with ATO during consolidation therapy, it is not possible to determine the independent contribution of isotretinoin to the observed molecular remission. The favorable outcome in this case may therefore largely reflect the therapeutic effect of ATO rather than isotretinoin [19,20].

Several additional limitations should be acknowledged. First, there is insufficient clinical data to determine the long-term relapse rates and survival outcomes associated with isotretinoin-based consolidation regimens compared to standard ATRA-based therapy. The existing literature consists primarily of isolated case reports rather than systematic studies. Second, optimal dosing strategies for isotretinoin in APL remain unclear due to variability among reported cases. Third, this is a single case report and cannot establish equivalence or non-inferiority to standard therapy. The favorable outcome may not be generalizable to all patients, particularly those with high-risk disease or those who have not achieved hematologic remission with standard induction therapy. Recent reviews continue to emphasize ATRA-based differentiation therapy as the cornerstone of treatment for APL [21,22].

## Conclusions

This case demonstrates that isotretinoin may achieve molecular remission in APL when used for consolidation therapy in combination with ATO. However, the independent contribution of isotretinoin to the observed remission cannot be determined, as the patient had already achieved hematologic remission with standard ATRA-based induction and received concurrent ATO, which has well-established single-agent efficacy in APL. Isotretinoin may have provided supplementary retinoid activity during consolidation, but its precise therapeutic contribution remains uncertain. Nonetheless, this case raises the important question of whether isotretinoin may serve as a temporary retinoid substitute in resource-limited settings when ATRA is inaccessible. While ATRA remains the evidence-based standard of care, the use of isotretinoin in this setting is non-standard and investigational. Although isotretinoin may represent a more affordable alternative, this has not been formally studied. Clinicians considering this approach should ensure close molecular monitoring with PML::RARA RT-PCR and maintain readiness to transition to standard therapy if available. Prospective studies are needed to establish optimal dosing, long-term efficacy, and safety of isotretinoin-based consolidation regimens.
